# Wechsler Intelligence Scale for Adults – Fourth Edition profiles of adults with autism spectrum disorder

**DOI:** 10.1017/S2045796022000506

**Published:** 2022-09-23

**Authors:** G. Cicinelli, E. Nobile, S. Brighenti, S. Bari, E. Tonella, A. Aresi, M. Attanasio, M. Mazza, M. Valenti, R. Keller

**Affiliations:** 1 Adult Autism Center, Mental Health Department, Local Health Unit ASL Città di Torino, Turin 10138, Italy; 2 Regional Centre for Autism, Dipartimento di Scienze cliniche applicate e biotecnologiche, University of L'Aquila, L'Aquila, Italy; 3 Department of Psychology, University of Turin, Via Giuseppe Verdi, 8, 10124 Torino, TO, Italy

**Keywords:** Autism spectrum disorder, intelligence, intelligence profile, WAIS-IV

## Abstract

**Aim:**

In this study, we have compared 229 Wechsler Adults Intelligence Scale – Fourth Edition (WAIS-IV) cognitive profiles of different severity adults with autism spectrum disorder to verify the impact of several variables including sex, age, level of education and autism severity level in an Italian sample. Moreover, we wanted to find out the optimal cut points for the major intelligence quotients in order to discriminate autism severity levels.

**Methods:**

Participants were recruited from two National Health System Center in two different Italian regions and were assessed with gold-standard instruments as a part of their clinical evaluation. According to DSM-5, cognitive domains were also measured with multi-componential tests. We used the Italian adaptation of WAIS-IV. We checked our hypotheses using linear regression models and receiver operating characteristics (ROC) curves.

**Results:**

Our results showed that age and level of education have a strong impact on Verbal Comprehension (VCI) and Working Memory Indexes (WMI). Gender differences are relevant when considering the VCI and Processing Speed index (PSI) in which women obtained the best performance. These differences are still relevant when considering cut points of ROC because 69 resulted to be the optimal cut point for women, 65 for men.

**Conclusions:**

Few conclusions can be assumed only examining Full Scale Intelligence Quotient (FSIQ) scores as it includes many different information about broader cognitive abilities. Looking deeper at main indexes and their subtests findings are consistent with previous research on the disorder (moderate correlations of FSIQ, Perceptual Reasoning index, WMI and PSI with the participants’ age), while other results are unforeseen (no effect of sex found on FSIQ score) or novel (significant effect of education on VCI and WMI). Using an algorithm predicting optimal cut point for discriminating through autism severity levels can help clinicians to better label and quantify the required help a person may need, a test cannot replace diagnostic and clinical evaluation by experienced clinicians.

## Introduction

Autism spectrum disorder (ASD) is a neurodevelopmental disorder with an early onset and a genetic component. ASD is characterised by deficits in socio-emotional reciprocity, impaired verbal and non-verbal communication skills and inability to develop and maintain adequate social relationships with peers. ASD core symptoms are associated with the presence of repetitive verbal and motor behaviours, restricted patterns of interest, need for an unchanging environment (or in any case predictable and stable) and hypo- or hypersensitivity to sensory inputs. The onset of clinical symptoms occurs during the early years of life (APA, 2013). Specifiers consider the possibility of several comorbidities, such as a cognitive deficit, language impairment, catatonia, medical or environmental factors or other neurodevelopmental disorders.

Recent prevalence estimates indicate 1 : 44 children in USA and 1 : 77 children in Italy (Maenner *et al*., 2016). Adults' prevalence is around 1 : 68 revealing a significant increase in the population of adults with ASD (Christensen *et al*., 2016). Alongside with this factor, another relevant element to be considered is the gender ratio across autistic people (Loomes *et al*., 2017) which is still debate and evidence mixed results. Sex-linked genetic factors and male vulnerability to brain insult may account for some of the gender differences (APA, 2013). Recent epidemiological studies revealed a 2–3 : 1 male-predominance compared to the widely cited 4–5 : 1 ratio from earlier studies (Mattila *et al*., 2011; Idring *et al*., 2012; Baxter *et al*., 2015; Zablotsky *et al*., 2015; Keller *et al*., 2020) although this ratio may depend upon intellectual abilities and it appears as low as 2 : 1 when ASD is associated with intellectual disability, and as high as 6–8 : 1 in high-functioning autism (HFA; Fombonne, 2005, 2009). It is supposed that this higher male prevalence is due to autistic females' abilities to mask their social difficulties, to cultural factors, a smaller number of studies on ASD in female population (Attwood, 2007; Lai *et al*., 2011; Kirkovski *et al*., 2013) and different ASD phenotypes (Mandy *et al*., 2012; Van Wijngaarden-Cremers *et al*., 2014; Howe *et al*., 2015). A recent study by Wilson *et al*. (2016) involving 1244 adults (935 males and 309 females) referred for ASD assessment reported sex differences in clinical outcome. Results concluded that 639 males and 188 female people were diagnosed with an ASD of any subtype. Indeed, in the study no significant effect of sex (male IQ > female IQ; *F*_(2)_ = 2.47, *p* = 0.09, *η*^2^*_p_* = 0.02) on IQ was found. Regarding intelligence outcomes, their results confirmed previous research reporting lower IQ score in females with a diagnosis of ASD compared to male participants (Fombonne, 2005). Indeed, Halpern and LaMay (2000) found no significant sex difference for the *g*-factor whereas sex differences play a role regarding achievements on subtests and indexes level using the Wechsler Intelligence Scale for Adults – 4th Edition (WAIS-IV; Wechsler, 2013).

Studies on typical developmental (TD) population examining the gender differences using subtests and derived indices from WAIS-IV highlighted better performances of men in IQ, Verbal Comprehension (VC), Perceptual Reasoning (PR) and Working Memory (WM) indices (Longman *et al*., 2007; Irwing, 2012; Daseking *et al*., 2017). Instead, Processing Speed (PS) index was the only in which women had better outcomes. These results were in line with an Italian study by Pezzuti *et al*. (2020) that found that men performed significantly better than women in the Arithmetic subtest and the WMI of the WAIS-IV. In their study comparing performances of TD on WAIS-R and WAIS-IV, gender differences appeared broader and more extensive in the WAIS-R sample, as other previous authors mentioned using WAIS-III (Dolan *et al*., 2006; Van der Sluis *et al*., 2006). A factor analysis study from Colom and Garcia-Lopez (2002) outlined that there are no sex differences in general ability (*g*) on the Spanish standardisation of the WAIS-III. Authors stated that the average sex differences favouring males have to be attributed to specific group factors and test specificity. Likewise, results obtained by Van der Sluis *et al*. (2006) using Dutch WAIS-III indicate differences between men and women in performance regarding specific cognitive abilities, but not in general intelligence (*g*). In contrast, for the US-standardisation sample of the WAIS-III, Irwing (2012) reported sex differences not only regarding specific abilities, but also in *g*. Men outperformed women in general intelligence [Full Scale Intelligence Quotient (FSIQ)] and on subtests like Information, Arithmetics and Symbol-Search, whereas women outperformed men on the Processing Speed Index (PSI).

Educational level (Ceci and Williams, 1997; Gustafsson, 2001) and age also contribute to the understanding of differences in IQ outcomes. Ceci (1991) suggests that the more years of education the better cognitive skills. This phenomenon is due to exposition of contexts that allow people to learn relevant information, to concentrate on problems, and it teaches approaches of cognition on which the majority of intelligence tests are based on. Results from an Italian study (Tommasi *et al*., 2015) showed that the WAIS-R detects individual differences in intelligence properly measured by IQ scores at different educational levels. Indeed, there is an average increase equal to 1.9 IQ points in the IQ global composite score per year of education. As previously hinted, age need to be considered when accounting for IQ differences and efficiency across time (Baltes *et al*., 1998; Schaie and Willis, 2010). Most of the studies focused on the key role of Working Memory and its connection to general abilities. It has been argued that in TD a significant detrimental effect of age on Working Memory resources is played (Craik and Salthouse, 2008; Robert *et al*., 2009).

So the profile of intelligence level is one of the relevant factors to be considered when diagnosing people with ASD, alongside with other cognitive, neuropsychological, socio-demographic and core-symptoms measures (Happé *et al*., 2016). Recognising how people with ASD may vary on this construct it may be crucial for identifying ASD subtypes (Grzadzinski *et al*., 2013). Therefore, ASD subtypes change according to different cognitive abilities’ patterns (Grzadzinski *et al*., 2013). Nonetheless there are no distinctive IQ profiles of individuals with ASD (Siegel *et al*., 1996; Ghaziuddin and Mountain-Kimchi, 2004; Goldstein *et al*., 2008; Williams *et al*., 2008; Charman *et al*., 2011). Intellectual abilities have been more challenging to assess in individuals with ASD because of their characteristics and assessment tools. Many researchers focused on children, few authors studied cognitive performances’ pattern in adults with ASD and how these patterns can differentiate severity levels and typical performance configurations. WAIS-IV (Wechsler, 2013) is the most widely used and renewed cognitive performance test for the assessment in verbal adults with ASD. Other standardised measures of intelligence include the Stanford–Binet (e.g. Roid, 2003), Raven's Progressive Matrices (RPM; Raven *et al*., 1998) and Leiter-3 (Roid *et al*., 2013). The use of Wechsler scales has been supported by several studies (Filipek *et al*., 1999; Mottron, 2004). Nevertheless, previous research has highlighted how the RPM (Raven *et al*., 1998) could be more adequate for describing the cognitive profile of people with ASD (Dawson *et al*., 2007; Hayashi *et al*., 2008; Soulières *et al*., 2011). Indeed, as pointed out by Dawson *et al*. (2007) the Wechsler scales may underestimate the intelligence of individuals with ASD mainly because of its emphasis on verbal instruction and tasks. However the structure and the characteristics of the RPM, suitable for fluid reasoning tasks, may be a more appropriate measure intelligence of people with ASD. Results of comparison between performances of Wechsler and RPM scores of adults with and without ASD highlighted a significantly higher performance of ASD group on RPM compared to TD group, whose performances across the scales were without significant differences. However, the IQ discrepancy between people with ASD and TD made uneasy the in-depth comprehension of the differences of the cognitive performances in ASD people using RPM and Wechsler scale. Results of a separate but related study suggest that the higher performance on the RPM as compared to the Wechsler measures primarily occurs for individuals with ASD with cognitive impairment (Bölte *et al*., 2009). Holdnack *et al*. (2011) compared performances between control group, HFA and Asperger disorder (AS) in the WAIS-IV subtests. No statistically significant differences between AS and control groups were found whereas the HFA group had the lowest scores. However, both ASD and control groups’ performances on Matrix Reasoning and Digits Forward revealed no significant differences. Regarding Coding subtests all three groups differed significantly from each other. Eventually, in Visual Puzzles where the HFA group performed significantly more poorly than the control group, the AS group did not differ from either the HFA or the control group.

Summing up, several demographic variables are associated with different cognitive level abilities in TD. However, based on our knowledge, no study evaluated together the effects of age, sex, level of education and level of autism on cognitive performances of people with ASD measured with the Italian WAIS-IV in a large sample. So that, in the present study we tested several hypotheses:

1. Test the association between the demographic variables and level of autism with FSIQ, main indexes and subtests, as a preliminary step for further and in-depth analyses. A moderate correlation with age and level of education and FSIQ and the main indexes was expected.
2. Assuming the FSIQ could not thoroughly explain strengths and weaknesses of people with ASD assessed with the WAIS-IV, we wanted to identify if like TD, significant effects of the independent variables were found on the four indexes together (VCI, WMI, PRI, PSI) and the underlying subtests. Specifically, we expected no sex differences in FSIQ in both level of autism; significant effects of age and level of education on VCI, WMI and PSI; ASD female participants' better performances on PSI.
3. Eventually, we wanted to test the hypothesis that better performances on the four indexes can predict less severe autistic symptoms. Indeed, optimal cut-off score for discriminate autism severity levels using WAIS-IV were investigated.

## Methods

### Participants

In total, 270 adults with ASD (*M*_age_ = 26.3 s.d. = 9.35) were evaluated in the Regional Center for Autism Spectrum Disorder in Turin and the Regional Centre for Autism in L'Aquila (Italy). The Regional Center of ASL Citta di Torino is a national mental health system department providing services for people with ASD. The centre provides clinical assessment, psychological and educational interventions for people with autism (Keller *et al*., 2020). The Regional Reference Center for Autism – a structure of Abruzzo Region Health System – performs diagnostic, clinical and consulting activities and provides treatments for individuals with ASD. Most of patients were referred by the general psychiatrist for an ASD assessment and came to either centre for the first time or returned for a follow-up evaluation. All the diagnoses were made according to Diagnostic and Statistical Manual of Mental Disorders, Fifth Edition (DSM-5) (APA, 2013) criteria considering clinical anamnesis, clinical interview, cognitive assessment with WAIS-IV (Orsini and Pezzuti, 2013), diagnostic evaluation with ADI-r (Rutter *et al*., 2003) and ADOS module 4 (Lord *et al*., 2002) or RAADS (Ritvo *et al*., 2011), following structured diagnostic pathway (multistep network model, Keller *et al*., 2020). Of the entire sample, 169 people received diagnosis of ASD with level 1 (male = 75%, *M*_edu_ = 12.4, s.d. = 2.64; female = 25%, *M*_edu_ = 13.6, s.d. = 2.91), 60 with ASD level 2 (male = 75%, *M*_edu_ = 10.9, s.d. = 2.18; female = 25%, *M*_edu_ = 11.3, s.d. = 2.47) and 39 with ASD level 3 (male = 79%, *M*_edu_ = 10.9, s.d. = 1.96; female = 21%, *M*_edu_ = 11.5, s.d. = 1.60). To be included in the study, all the patients received a formal clinical diagnosis of ASD according to DSM-5 (APA, 2013) criteria. People with comorbid psychopathology (*n* = 42) were included only if is either in remission or of minimal impact on daily functioning. In total, 3.9% with ASD level 1 and comorbid depressive disorders (male = 3%, female = 0.9%), 3.49% with ASD level 1 and personality disorders (male = 2.18%, female = 1.31%), 2.18% with ASD level 1 and specific learning disorders (male = 1.31%, female = 0.87%), 1.31% people with ASD level 1 (male = 0.43%, female = 0.86%) and 0.43% males with ASD level 2 and obsessive-compulsive disorder, 1.31% with ASD level 1 and epilepsy (male = 0.87%, female = 0.43%), 1.31% with ASD level 1 and anxiety disorder (male = 0.43%, female = 0.87%), 1.31% with ASD level 1 and schizophrenia (male = 0.87%, female = 0.43%), 0.87% with ASD level 1 and attention-deficit/hyperactivity disorder (male = 0.43%, female = 0.43%), 0.87% with ASD level 1 and developmental coordination disorder (male = 0.43%, female = 0.43%), 0.43% females with ASD level 1 and Turner syndrome, 0.43% males with ASD level 2 and Tourette syndrome, 0.43% with ASD level 1 and gender dysphoria were included.

In total, 39 participants with level 3 and two participants with level 2 were excluded from the original sample because they resulted not suitable for a verbal cognitive evaluation with WAIS-IV since their communication was through gestures or other alternative communication systems.

All demographic variables and characteristics of the final sample are presented in Table 1.

**Table 1.** Demographic characteristics of the sample (total *n* = 229)

	ASD level	Age	Education
Mean	1	27.3	12.7
	2	23.5	11.0
Median	1	23	13
	2	21.0	12.0
Standard deviation	1	10.1	2.75
	2	6.09	2.24
Minimum	1	16	8
	2	18	5
Maximum	1	64	21
	2	51	13

### Measures

Data about cognitive abilities were collected using the WAIS-IV (Wechsler, 2013). The WAIS-IV is used to assess intellectual profile for people between 16 and 90 years old. It is composed by four scores and a general intelligence index. The four indexes are VCI, PRI, WMI and PSI. Every index is composed by two or three subtest that are required to obtain the total IQ score. The ten-core subtest are: Vocabulary, Information, Similarities, Digit Span, Arithmetic, Block Design, Matrix Reasoning, Visual Puzzles, Coding and Symbol Search. It also contains five additional subtests: Comprehension, Letter–Number Sequencing, Figure Weights, Picture Completion and Cancellation. In our sample we used the ten-core subtests for all ASD people and levels. We calculated the subtests scores, the indexes’ scores and the full-scale IQ index. Every raw score was corrected with Italian standardisation scores of the WAIS-IV (Orsini and Pezzuti, 2013).

The WAIS-IV and the entire psychological evaluation was administered by licensed psychologist in a large and bright room in one session from 45 min to 1.5 h.

The structure of the WAIS-IV and its indexes and subtests is represented in Table 2.

**Table 2.** Full Scale and Primary Index scales

Full Scale			
Verbal Comprehension	Perceptual Reasoning	Working Memory	Processing Speed
Similarities Vocabulary Information *Comprehension*	Block Design Matrix Reasoning Visual Puzzles *Figure Weights* *Picture Completion*	Digit Span Arithmetic *Letter-Number Sequencing*	Symbol Search Coding *Cancellation*

*Note*: Italics indicate supplemental subtests.

Age of each participant was calculated at the moment of the WAIS-IV administration and expressed in integers.

Level of autism was classified in three different levels as stated in DSM-5 (APA, 2013), so that level 1 was the less severe while level 3 the most severe. Level of severity was assessed through clinical interviews made by two independent psychologists and a psychiatrist with participants and caregivers. Eventually, in a final reunion the entire professional team discussed and agreed for one of the three levels of support required by the person.

Years of educations were collected considering each school cycle years entirely completed. Any interrupted instruction years was not added to the number. Thus, considering Italian compulsory education system, 5 years were assigned if a person completed the first school cycle. Other 3 years were given if a person completed the second school cycle. Finally, 5 years were considered if a person completed the last compulsory education cycle. Moreover, 3 to 5 years of additional educational years were given if a person completed a bachelor or a master's degree.

Psychopathological comorbidity was considered as a dichotomous variable in terms of the presence or absence of any disorder.

### Data analysis

Analytical approach was used to better describe and understand data collected. At first, descriptive and correlational analysis were run to explore data and the distribution of the variables across ASD levels and to determine whether there was a relationship between the variables of interest. A moderate association between variables represents one of the conditions for exploring cause–effect phenomena through in-depth subsequent analysis.

Indeed, to better understand the effects of socio-demographic and ASD-related variables on cognitive performance indexes, linear regression models were used to analyse the impact of age, education, ASD level, sex and comorbidity on WAIS-IV indexes. Linear regression is a predictive analysis used to determine if a set of predictor variables (independent variables) predict an outcome (dependent variables). Through analysis of variance test we evaluated an ‘overall’ effect considering the differences between means. Instead, the *p*-value for each mean in the regression models was used to easier understand which mean is different from the reference one.

Moreover, in a cascade approach model, we performed a more in-deep analyses considering each index as a dependent variable and socio-demographic and ASD-related variables as covariates. For the subsequent analyses we performed a multivariate analysis of covariance (MANCOVA) to assess for statistical differences on multiple continuous dependent variables – the four WAIS-IV indexes – by two independent grouping variables, while controlling for one or more variables called the covariates. Through MANCOVA we created a model with four dependent variables (the four WAIS-IV indexes), sex, ASD-level and comorbidity as independent variables and age and education as covariates. Eventually, we repeated the same analysis using each indexes' subtests as dependent variable, sex, ASD-level and comorbidity as independent variables and age and education as covariates.

Likewise, consistent with the third aim of the research, we wanted to discriminate among ASD-severity levels. Area under the curve (AUC) and receiver operating characteristics (ROC) (Metz, 1978; Zweig and Campbell, 1993) were used to inspect the performance of the two ASD-level groups on WAIS-IV composite indexes. ROC–AUC reveals how much the five WAIS-IV composite scores are capable of distinguishing between ASD-severity levels. The higher the AUC, the better the model is at distinguishing between participants with 1 and 2 severity levels. An ROC is a plot of the true-positive rate (sensitivity) *v*. false-positive rate (1-specificity) associated with every possible cut-off value for a measure. The AUC is a measure of diagnostic accuracy and predictive validity that can be used to compare the predictive value of different measures. The AUC can range between 0.5 (random discrimination) and 1 (perfect discrimination).

For the analysis we used R Studio (R Studio Team, 2020) and Jamovi (The Jamovi Project, 2021) software.

## Results

**Fig. 1.**
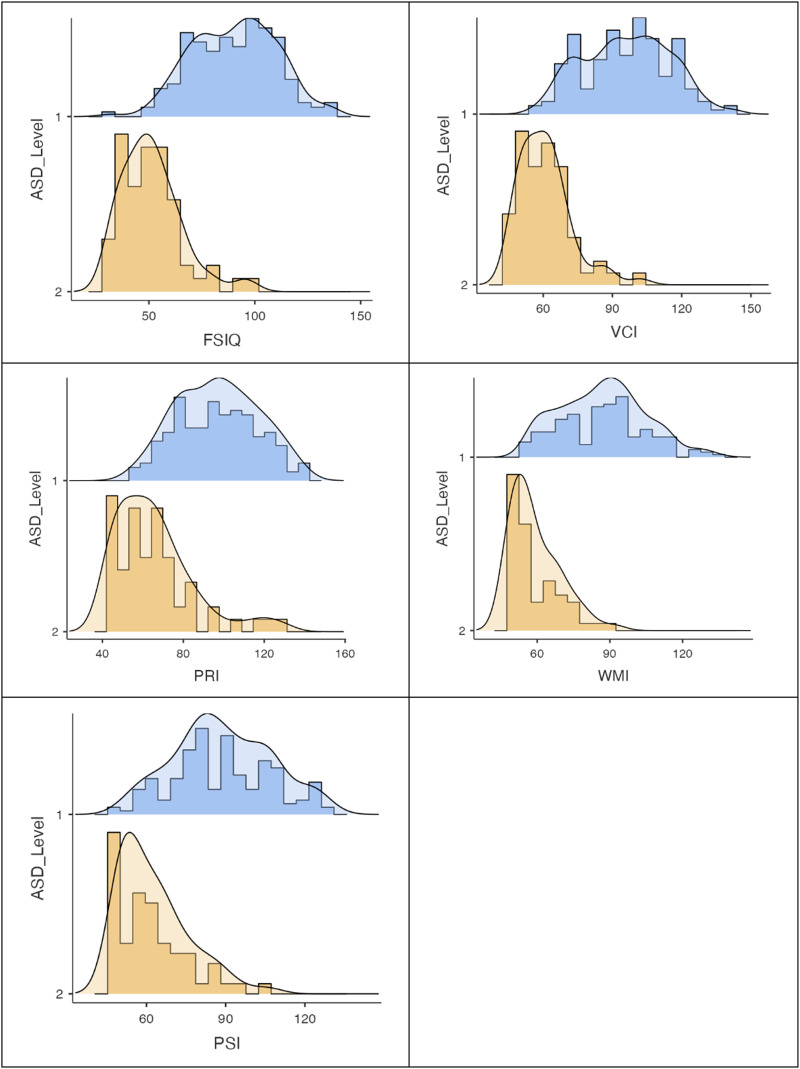
Histograms with density of Full Scale and Primary Index scales on different ASD-level groups. *Note*: ASD_Level, level of autism; FSIQ, Full Scale Intelligence Quotient; VCI, Verbal Comprehension Index; PRI, Perceptual Reasoning Index; WMI, Working Memory Index; PSI, Processing Speed Index.

For statistical analysis, two adults with level 2 and 39 adults of level 3 were excluded because they could not be assessed with the WAIS-IV. So, the final sample was composed by 229 people of level 1 and 2. The descriptive statistics of the sample and the four indexes are presented in Table 3. For better understanding data distribution across the levels and indexes we presented histograms with density of the FSIQ and the four indexes in Fig. 1.

**Table 3.** Descriptive statistics of the sample

	ASD level	FSIQ	VCI	PRI	WMI	PSI
Mean	1	90.5	96.1	97.0	86.9	88.9
	2	51.3	60.8	65.5	59.1	62.7
Median	1	92	96	96	89	89
	2	49	61.0	62	57.0	58.0
Standard deviation	1	19.9	18.9	20.1	17.6	18.5
	2	14.2	11.2	19.5	10	13.2
Minimum	1	32	55	54	55	50
	2	32	47	44	49	50
Maximum	1	137	143	139	134	131
	2	97	102	129	92	106

In simple correlation analysis (see Table 4), age was significantly correlated with FSIQ (*r* = 0.300, *p* < 0.001), VCI (*r* = 0.323, *p* = 0.01), PRI (*r* = 0.214, *p* = 0.001), WMI (*r* = 0.247, *p* < 0.001) and PSI (*r* = 0.235, *p* < 0.001). A relevant result was the absence of significance between block design and age (*r* = 0.084, *p* = 0.207). Similar result was found between Arithmetic and age (*r* = 0.206; *p* = 0.002). Level of education was significantly correlated with FSIQ (*r* = 0.376, *p* < 0.001), while the stronger association was only with the VCI (*r* = 0.264, *p* < 0.001) and its subtests, Similarities (*r* = 0.346, *p* < 0.001), Vocabulary (*r* = 0.387, *p* < 0.001) and Information (*r* = 0.366, *p* < 0.001). Although no significant correlation between level of education and WMI was found, Arithmetic was moderately correlated with level of education (*r* = 0.301; *p* < 0.001).

**Table 4.** Correlation Matrix of Full Scale, Primary Index Scales and main subtests

	Age	Edu	FSIQ	PRI	VCI	WMI	PSI	BD	SI	DS	MR	VC	AR	SS	VP	IN	CO
Age	–																
Edu	0.310***	–															
FSIQ	0.300***	0.376***	–														
VCI	0.323***	0.394	0.889***	–													
PRI	0.214**	0.264***	0.898***	0.701***	–												
WMI	0.247***	0.328***	0.848***	0.722***	0.691***	–											
PSI	0.235***	0.275***	0.821***	0.631***	0.707***	0.632***	–										
BD	0.084	0.153*	0.580***	0.450***	0.673***	0.455***	0.498***	–									
SI	0.229***	0.346***	0.779***	0.887***	0.634***	0.618***	0.521***	0.450***	–								
DS	0.229***	0.303***	0.708***	0.598***	0.548***	0.906***	0.518***	0.322***	0.527***	–							
MR	0.201**	0.247***	0.838***	0.689***	0.860***	0.720***	0.646***	0.537***	0.644***	0.610***	–						
VC	0.321***	0.387***	0.800***	0.903***	0.602***	0.652***	0.605***	0.353***	0.766***	0.588***	0.594***	–					
AR	0.206**	0.301***	0.833***	0.706***	0.714***	0.914***	0.625***	0.524***	0.642***	0.710***	0.722***	0.655***	–				
SS	0.242***	0.260***	0.764***	0.594***	0.670***	0.561***	0.914***	0.446***	0.516***	0.504***	0.597***	0.603***	0.565***	–			
VP	0.203**	0.211**	0.813***	0.645***	0.911***	0.602***	0.640***	0.592***	0.592***	0.485***	0.709***	0.582***	0.643***	0.646***	–		
IN	0.287***	0.366***	0.820***	0.891***	0.674***	0.666***	0.559***	0.399***	0.721***	0.568***	0.653***	0.767***	0.687***	0.550***	0.623***	–	
CO	0.232***	0.292***	0.760***	0.575***	0.648***	0.610***	0.915***	0.385***	0.492***	0.550***	0.646***	0.579***	0.592***	0.794***	0.580***	0.525***	–

Edu, level of education; FSIQ, Full Scale Intelligence Quotient; VCI, Verbal Comprehension Index; PRI, Perceptual Reasoning Index; WMI, Working Memory Index; PSI, Processing Speed Index; BD, Block Design; SI, Similarities; DS, Digit Span; MR, Matrix Reasoning; VC, Vocabulary; AR, Arithmetic; SS, Symbol Search; VP, Visual Puzzles; IN, Information; CO, Coding.

*p < 0.05, **p < 0.01, ***p < 0.001.

All the associations between the main indexes and subtests were all significant (*p* < 0.001).

In linear regression models we considered the joint effects of sex, level of education, level of autism, age and comorbidity on FSIQ. In model 1, age (*β* = 0.371; *t* = 2.779; *p* = 0.006), level of autism (*β* = −35.205; *t* = −12.636; *p* < 0.001) and level of education (*β* = 1.530; *t* = 3.268; *p* < 0.001) were significant, suggesting that the higher the age, the level of autism and education, the better the FSIQ score. Model 1 explained 54.3% of variance in FSQI scores (*R*^2^ adjusted = 0.512, *F*_(4, 224)_ = 60.9, *p* < 0.001). No significant effects of comorbidity were found on FSIQ (*β* = 0.479; *t* = 0.153; *p* = 0.87).

Using multivariate multiple regressions models with MANCOVA we tested different hypotheses. In model 2 we considered the joint effects of the previous model independent variables separately on the four indexes (VCI, PRI, WMI, PSI). Sex (*F* = 8.23; *p* < 0.001), age (*F* = 4.54; *p* = 0.002), level of education (*F* = 3.53; *p* = 0.008) and level of autism (*F* = 63.80; *p* < 0.001) have a significant impact on the four indexes when considering them together. No significant effects were found considering the joint effects of sex and level of autism on the four indexes (*F* = 1.95; *p* = 0.103) nor of comorbidities (*F* = 1.77; *p* = 0.135). Therefore, model 2 suggests that male patients perform better than females and the higher the level of education and age the better the four indexes' scores. Indeed, considering the direct effect of the variables on every single index we found that the effect of sex was statistically significant on VCI (*F* = 4.429; *p* = 0.036) and PSI (*F* = 10.835; *p* = 0.001) and remain significant when the joint effect with level is considered on PSI (*F* = 6.788; *p* = 0.010). Education has a statistically significant effect on VCI (*F* = 12.374; *p* ⩽ 0.001) and WMI (*F* = 8.288; *p* = 0.004).

In the following multivariate multiple regressions models we evaluated the effects of sex, age, educational, autism levels and comorbidities on the core subtests of the four indexes. Digit Span and Arithmetic were considered as the core subtests of WMI. The results highlighted significant effect of level of autism (*F* = 73.036; *p* < 0.001), age (*F* = 3.832; *p* = 0.023) and education (*F* = 4.244; *p* = 0.016) on both subtests. No effects of comorbidities were found on WMI subtests (*F* = 0.121; *p* = 0.886).

Considering the core subtests of VCI, sex (*F* = 2.859; *p* = 0.038), level of education (*F* = 4.822; *p* = 0.003), level of autism (*F* = 73.258; *p* < 0.001) and age (*F* = 5.932; *p* < 0.001) had a statistically significant impact on Similarities, Vocabulary and Information. If we look at the univariate tests' results sex has a significant impact only on Vocabulary (*F* = 7.337; *p* = 0.007) with no significance on Similarities and Information. No effects of comorbidities were found on VCI subtests (*F* = 0.623; *p* = 0.601).

Indeed, for the effects on Block Design, Matrix Reasoning and Visual Puzzles, level of autism was the only covariate with a strong impact on the three subtests (*F* = 44.375; *p* < 0.001). No other relevant results were found except for a small significant effect of sex and autism levels on VP (*F* = 4.433; *p* = 0.036).

The last model considered the effects of variables on Symbol Search and Coding and revealed a significant effect of sex (*F* = 5.21; *p* = 0.006), level of autism (*F* = 60.29; *p* < 0.001) and the interaction between sex and level of autism (*F* = 3.22; *p* = 0.042) on the two subtests. However, when the effect of the variables isolated on each subtests age have a statistically significant impact on Symbol Search.

ROC results are presented in Table 5. According to the previous analysis, sex was statistically different on several indexes and subtest, and because of the small female sample size we decided to treat males and females separately. In Table 5 we used ROC on female (*n* = 57) and male (*n* = 172) samples. Different cut-points were found to be discriminative between level 1 and 2 considering FSIQ. Each index differed statistically significantly from chance level (*α* = 0.05).

**Table 5.** Predictive values for FSIQ and the four main indexes for the female sample (*n* = 57)

	FSIQ			VCI			PRI			WMI			PSI		
Cut point	Sensitivity (%)	Specificity (%)	AUC	Sensitivity (%)	Specificity (%)	AUC	Sensitivity (%)	Specificity (%)	AUC	Sensitivity (%)	Specificity (%)	AUC	Sensitivity (%)	Specificity (%)	AUC
69	97.62	100	0.995	–	–	–	–	–	–	92.86	100	0.990	–	–	–
72	–	–	–	–	–	–	–	–	–	–	–	–	92.86	80	0.956
73	95.24	100	0.995	–	–	–	–	–	–	–	–	–	–	–	–
74	92.86	100	0.995	100	93.33	0.990	–	–	–	–	–	–	–	–	–
76	–	–	–	95.24	93.33	0.990	–	–	–	–	–	–	–	–	–
78	–	–	–	–	–	–	–	–	–	–	–	–	88.1	86.67	0.956
79	–	–	–	––	–	–	88.1	93.33	0.952	–	–	–	–	–	–
81	–	–	–	–	–	–	83.33	93.33	0.952	–	–	–	83.33	93.33	0.956
86	–	–	–	–	–	–	–	–	–	–	–	–	78.57	93.33	0.956
Predictive values for FSIQ and the four main indexes for the male sample (*n* = 172)															
65	88.19	86.67	0.923	–	–	–	–	–	–	–	–	–	–	–	–
66	87.4	86.67	0.923	–	–	–	–	–	–	–	–	–	–	–	–
67	84.25	86.67	0.923	94.49	77.78	0.933	–	–	–	–	–	–	–	–	–
68	82.68	88.89	0.923	–	–	–	–	–	–	–	–	–	–	–	–
69	81.89	88.89	0.923	90.55	82.22	0.933	–	–	–	–	–	–	–	–	–
70	–	–	–	–	–	–	–	–	–	–	–	–	82.68	75.56	0.843
71	–	–	–	86.61	82.22	0.933	90.55	66.67	0.847	–	–	–	–	–	–
72	–	–	–	–	–	–	–	–	–	77.17	84.44	0.892	79.53	75.56	0.843
73	–	–	–	82.68	86.67	0.933	88.98	68.89	0.847	–	–	–	–	–	–
74	–	–	–	80.31	91.11	0.933	–	–	–	–	–	–	–	–	–
75	–	–	–	–	–	–	85.04	71.11	0.847	74.02	84.44	0.892	77.17	80	0.843
76	–	–	–	77.95	91.11	0.933	–	–	–	–	–	–	–	–	–
77	–	–	–	–	–	–	82.68	77.78	0.847	69.29	91.11	0.892	–	–	–
78	–	–	–	–	–	–	–	–	–	–	–	–	71.65	82.22	0.843
79	–	–	–	–	–	–	78.74	77.78	0.847	–	–	–	–	–	–
80	–	–	–	–	–	–	–	–	–	63.78	93.33	0.892	–	–	–
81	–	–	–	–	–	–	–	–	–	61.42	95.56	0.892	–	–	–

In the female sample, a score of 69 differentiates between levels while a range varying from 65 to 69 scores can distinguish between male with different autism levels. VCI distinguishes between level 1 and 2 at score 74 in female participants. Whereas, in male participants a clinical range to consider varies from 67 to 76. PRI's best score for female sample is 79 while for the male sample a score of 77 is the best compromise considering sensitivity and specificity. Regarding WMI, a cut-point of 69 resulted in a strong parameter for distinguishing level 1 and 2 autism in women. For male population an adequate cut-point is 72 with a good sensitivity and specificity. Finally, for the PSI, in the female sample 81 was a good cut-point, while for the male sample the good cut-point was 70.

## Discussion

Limited researchers focused on in-depth study of the cognitive profile of adults with autism in the international context and no research in the Italian context (Fombonne, 2005; Wilson *et al*., 2016). To our knowledge, the majority of authors focused on cognitive and social performances of children or adolescent with ASD (Bodner *et al*., 2014). Several studies focused on comparing cognitive performance of adults with ASD with TD or HFA with AS and TD (Holdnack *et al*., 2011). None of them explored the effect of socio-demographic variables on cognitive performances of people with ASD. So, in our research we explored the cognitive profile of adults with ASD that reached a clinical diagnosis. After exploring data with descriptive analyses, we performed a correlation of Full Scale, Primary Index Scales and main subtest and socio-demographic variables. The results showed that FSIQ, PRI, WMI and PSI moderately correlate with the age of the participants. More specifically, it is supposed that level of education has a significant impact on cognitive skills measured by WAIS-IV indexes (Ceci, 1991; Baltes *et al*., 1998; Schaie and Willis, 2010; Pezzuti *et al*., 2019; Borella *et al*., 2020). Instead, an interesting result is the almost independence of the subtest Block Design from age and education which can be considered as a culturally and age-independent subtest in our sample.

Subsequently, we used a cascade approach, analysing at first the Full-Scale Index, then the four fundamental indexes and eventually the subtests that form the four main indexes. The decision for this choice was made to reduce the impact of two errors: the errors made during the transformation of the weighted scores into composite scores and when the difference between the indexes or the subtests were such as to invalidate the score of the index itself. In the first linear regression model we evaluated the impact of age, level of education, sex and level of autism on the FSIQ. The results showed a high level of significance for both age and education, indicating that each score in the FSIQ is correlated with an increase of 0.37 years and, for each year of education there is an increase of approximately 1.5 points in the FSIQ. These results are in line with a study on TD by Tommasi *et al*. (2015) that evidenced an average increase of 1.9 IQ points in the IQ global composite score per year of education. Contrary to our expectations and previous results that evidenced autistic female disadvantage in IQ scores compared to autistic male, no sex effects were found on the FSIQ score in our sample. As previously mentioned, few conclusions can be assumed only examining FSIQ scores as it includes many different information about broader cognitive abilities.

Therefore, in model 2 we ran a MANCOVA using the four indexes as dependent variables, sex and severity levels as factors and age and education as covariates. The results showed a statistically significant difference in all the variables except when the interaction between sex and level of autism is considered. Looking deeper at results and the impact of the variables on indexes, results highlight a significant sex difference on Verbal Comprehension and Processing Speed indexes in the female participants which perform better than the male peers. This latter result is not surprising since even TD female adults outperformed male in processing speed tasks (Daseking *et al*., 2017). However, unexpectedly, and never outlined before, female autistic adults had better performances in vocabulary compared to autistic males. Although these results are surprising and new, further studies needed to be conducted counterbalancing the number of female and male ASD participants. The effect of female advantage on PSI remains significant when the interaction with ASD level is considered. Indeed, the performance of female participants on PSI is better both in ASD level 1 and 2. Another not surprisingly result is the effect of education on Verbal Comprehension index suggesting that people with higher education perform better in verbal acquired knowledge and verbal reasoning, as previous literature pointed out (Tommasi *et al*., 2015). However the effects of education on Working Memory are partly new and remain significant when both subtests are considered for the analysis. However, further studies need to be conducted to better understand the direction of this effect. In fact, it can be postulate as years of education contribute to better Digit Span and Arithmetic performances as better WMI's performances increase the likelihood of a higher level of education. Unpredictably no statistical effect of sex on WM was found, revealing a similar way for both male and female participants to perform in this cognitive domain. This result is in contrast to a recent Italian study on TD by Pezzuti *et al*. (2020) in which there was an outperformance of men in WMI composite scores and its Arithmetic subtest. The absence of effects of sex on this index in our autistic sample could be interpreted in light of extreme male brain theory (Baron-Cohen, 2002) whereby autism can be considered as an extreme of the normal male profile.

In model 4 subtests of the VCI (Similarities, Vocabulary and Information) are considered and the results showed a significant effect of all the variables except when the interaction between sex and ASD level is taken into account. Looking deeper at the univariate analyses, the significant effects of education, age and level of autism on individual subtests are confirmed on each subtest. The literature supports these findings, showing that the level of education is a predictor of greater verbal competence (Abad *et al*., 2015). However, the previous sex differences found considering the VCI composite scores disappeared when each subtest is considered for the analysis, except for Vocabulary. Even this result is in contrast to previous research (Longman *et al*., 2007; Irwing, 2012; Daseking *et al*., 2017) that outlined superiority of men with TD in Verbal Comprehension Index. Conversely, in our sample females with ASD outperformed male with ASD when Vocabulary subtest is considered in the analysis. However, this difference is considered statistically significant only at ASD level 1, no sex differences in VCI subtests were detected when ASD level 2 is considered.

In model 5 we used the subtests Block Design, Matrix Reasoning and Visual Puzzles as dependent variables. The results showed only a significant effect of the level of ASD on the subtests considered. Superiority of male with TD in PRI composite score (Longman *et al*., 2007; Irwing, 2012; Daseking *et al*., 2017) was not confirmed in our autistic sample, indicating that subtests of PRI are more sensitive to ASD severity level in our sample.

In model 6, Symbol Search and Coding were used as dependent variables. The results revealed a statistically significant effect of sex and levels of autism on both subtests, confirming the previous results when the PSI composite score was analysed. Even when the joint effect of sex and level of autism is controlled, the result remains statistically significant on each subtest. This result is in line with the previous studies on TD considering the female superiority in Processing Speed Index (Pezzuti *et al*., 2020); hence the same pattern seems to occur in ASD population.

Using WAIS-IV main indexes or subtest cut-off scores to better discriminate between autism levels' can be controversial but useful for clinicians who must describe one person functioning according to DSM-5 (APA, 2013) classification. For the Full-Scale Index, the best cut-points revealed were 69 for female and 65 for male using Youden's indexes. In VCI, the optimal cut-points were 74 and 69 for females and males, respectively; regarding the PRI, the best cut-points were 79 for females and 73 for males; in WMI 69 for females and 72 for males; finally, for PSI the optimal cut-points were 81 for females and 70 for males.

Although all these predictive results can help clinicians to better discriminate between different levels of severity, a test cannot replace diagnostic assessment by experienced clinicians. However, cut-off scores taken together with the previous findings about the almost independency of PRI from age, level of education and sex can partly direct clinical evaluation to visuo-spatial abilities when assessing people with ASD across levels.

To sum up, some authors evidenced an underestimating effect of cognitive abilities of ASD people when assessed with WAIS-IV compared to RPM (Dawson *et al*., 2007; Hayashi *et al*., 2008; Soulières *et al*., 2011). However, this phenomenon seems to better apply to ASD people with cognitive impairment and not to AS (Bölte *et al*., 2009; Holdnack *et al*., 2011) or average cognitive abilities. So, cognitive impairment should be of concern when selecting any assessment tool to use with people with ASD and when interpreting results of their achievement on that measure. Alongside with cognitive impairment, language delay plays a significant impact on IQ outcome, as Bodner *et al*. (2014) evidenced in their study that resulted in better WAIS-IV IQ than RPM scores in verbally able adults. Thus, multiple factors need to be considered before assessing people with ASD (context, situation, abilities assessed, different methods) prioritising a multi-method multi-informant approach. Therefore, predicting academic or adaptive functioning of people with ASD across life-span based on cognitive assessment tools should be done with caution since neither the Wechsler nor the RPM fully gather all the information need to assess cognitive functioning in people with ASD.

### Limitations and directions for future research

A possible limitation of the study is the small number of female participants compared to the male participants, which may preclude the generalisation of results. Besides, the reduced female ASD sample and the results of no sex differences on IQ general composite scores can be partly due to female sample size. However, the sample was composed by different numbers of males and females according to the ASD prevalence.

Only the presence or absence of comorbidities on findings has been investigated in the research. Although a limited number of participants had clinical diagnosis that could have a strong effect on WAIS-IV subtests, as Psychotic Disorders or ADHD, further studies are needed to evaluate the single effect of comorbidities on outcomes.

## Availability of data and materials

The anonymised datasets analysed in the current study are available from the corresponding author on request.

## References

[ref1] Abad F, Sorrel M, Román F and Colom R (2015) The relationships between WAIS-IV factor index scores and educational level: a bifactor model approach. Psychological Assessment 28, 987–1000. 26322798 10.1037/pas0000228

[ref2] American Psychiatric Association (2013) Diagnostic and Statistical Manual of Mental Disorders, 5th Edn. Arlington, VA: Author.

[ref3] Attwood T (2007) The Complete Guide to Asperger's Syndrome. London: Jessica Kingsley Publishers.

[ref4] Baltes PB, Lindenberger U and Staudinger UM (1998) Life span theory in developmental psychology. In Damon W and Lerner RM (eds), Handbook of Child Psychology: Vol. 1. Theoretical Models of Human Development, 5th Edn. Hoboken, NJ: Wiley, pp. 1029–1143.

[ref5] Baron-Cohen S (2002) The extreme male brain theory of autism. Trends in Cognitive Sciences 6, 248–254. 12039606 10.1016/s1364-6613(02)01904-6

[ref6] Baxter AJ, Brugha TS, Erskine HE, Scheurer RW, Vos T and Scott JG (2015) The epidemiology and global burden of autism spectrum disorders. Psychological Medicine 45, 601–613. 25108395 10.1017/S003329171400172X

[ref7] Bodner KE, Williams DL, Engelhardt CR and Minshew NJ (2014) A comparison of measures for assessing the level and nature of intelligence in verbal children and adults with autism spectrum disorder. Research in Autism Spectrum Disorders 8, 1434–1442. 25180048 10.1016/j.rasd.2014.07.015 PMC4148695

[ref8] Bölte S, Dziobek I and Poustka F (2009) Brief report: the level and nature of autistic intelligence revisited. Journal of Autism and Developmental Disorders 39, 678–682. 19052857 10.1007/s10803-008-0667-2

[ref9] Borella E, Pezzuti L, De Beni R and Cornoldi C (2020) Intelligence and working memory: evidence from administering the WAIS-IV to Italian adults and elderly. Psychological Research 84, 1622–1634. 30949787 10.1007/s00426-019-01173-7

[ref10] Ceci SJ (1991) How much does schooling influence general intelligence and its cognitive components? A reassessment of the evidence. Developmental Psychology 27, 703–722.

[ref11] Ceci SJ and Williams WM (1997) Schooling, intelligence, and income. American Psychologist 52, 1051.

[ref12] Charman T, Pickles A, Simonoff E, Chandler S, Loucas T and Baird G (2011) IQ in children with autism spectrum disorders: data from the Special Needs and Autism Project (SNAP). Psychological Medicine 41, 619–627. 21272389 10.1017/S0033291710000991

[ref13] Christensen DL, Baio J, Van Naarden Braun K, Bilder D, Charles J, Constantino JN, Daniels J, Durkin MS, Fitzgerald RT, Kurzius-Spencer M, Lee LC, Pettygrove S, Robinson C, Schulz E, Wells C, Wingate MS, Zahorodny W, Yeargin-Allsopp M and Centers for Disease Control and Prevention (CDC) (2016) Prevalence and characteristics of autism spectrum disorder among children aged 8 years – autism and developmental disabilities monitoring network, 11 sites, United States, 2012. MMWR Surveillance Summaries 65, 1–23. 10.15585/mmwr.ss6503a1 PMC7909709 27031587

[ref14] Colom R and Garcia-Lopez O (2002) Sex differences in fluid intelligence among high school graduates. Personality and Individual Differences 32, 445–451.

[ref15] Craik FIM and Salthouse TA (2008) Handbook of Cognitive Aging, 3rd Edn. New York: Psychology Press.

[ref16] Daseking M, Petermann F and Waldmann HC (2017) Sex differences in cognitive abilities: analyses for the German WAIS-IV. Personality and Individual Differences 114, 145–150.

[ref17] Dawson M, Souliéres I, Gernsbacher MA and Mottron L (2007) The level and nature of autistic intelligence. Psychological Science 18, 657–662. 17680932 10.1111/j.1467-9280.2007.01954.x PMC4287210

[ref18] Dolan CV, Colom R, Abad FJ, Wicherts JM, Hessen DJ and van de Sluis S (2006) Multi-group covariance and mean structure modeling of the relationship between the WAIS-III common factors and sex and educational attainment in Spain. Intelligence 34, 193–210.

[ref19] Filipek PA, Accardo PJ, Baranek GT, Cook EH Jr., Dawson G, Gordon B, Gravel JS, Johnson CP, Kallen RJ, Levy SE, Minshew NJ, Ozonoff S, Prizant BM, Rapin I, Rogers SJ, Stone WL, Teplin S, Tuchman RF and Volkmar FR (1999) The screening and diagnosis of autistic spectrum disorders. Journal of Autism and Developmental Disorder 29, 439–484. 10.1023/a:1021943802493 10638459

[ref20] Fombonne E (2005) Epidemiology of autistic disorder and other pervasive developmental disorders. Journal of Clinical Psychiatry 66, 3–8. 16401144

[ref21] Fombonne E (2009) Epidemiology of pervasive developmental disorders. Pediatric Research 65, 591–598. 19218885 10.1203/PDR.0b013e31819e7203

[ref22] Ghaziuddin M and Mountain-Kimchi K (2004) Defining the intellectual profile of Asperger syndrome: comparison with high-functioning autism. Journal of Autism and Developmental Disorders 34, 279–284. 15264496 10.1023/b:jadd.0000029550.19098.77

[ref23] Goldstein G, Allen D, Minshew N, Williams D, Volkmar F, Klin A and Schultz RT (2008) The structure of intelligence in children and adults with high functioning autism. Neuropsychology 22, 301–312. 18444708 10.1037/0894-4105.22.3.301 PMC3093048

[ref24] Grzadzinski R, Huerta M and Lord C (2013) DSM-5 and autism spectrum disorders (ASDs): an opportunity for identifying ASD subtypes. Molecular Autism 4, 12. 23675638 10.1186/2040-2392-4-12 PMC3671160

[ref25] Gustafsson JE (2001) Schooling and intelligence: effects of track of study on level and profile of cognitive abilities. International Education Journal 2, 166–186.

[ref26] Halpern DF and LaMay ML (2000) The smarter sex: a critical review of sex differences in intelligence. Educational Psychology Review 12, 229–246.

[ref27] Happé FG, Mansour H, Barrett P, Brown T, Abbott P and Charlton RA (2016) Demographic and cognitive profile of individuals seeking a diagnosis of autism spectrum disorder in adulthood. Journal of Autism and Developmental Disorder 46, 3469–3480. 10.1007/s10803-016-2886-2 27549589

[ref28] Hayashi M, Kato M, Igarashi K and Kashima H (2008) Superior fluid intelligence in children with Asperger's disorder. Brain and Cognition 66, 306–310. 17980944 10.1016/j.bandc.2007.09.008

[ref29] Holdnack J, Goldstein G and Drozdick L (2011) Social perception and WAIS-IV performance in adolescents and adults diagnosed with Asperger's syndrome and autism. Assessment 18, 192–200. 21220381 10.1177/1073191110394771

[ref30] Howe YJ, O'Rourke JA, Yatchmink Y, Viscidi EW, Jones RN and Morrow EM (2015) Female autism phenotypes investigated at different levels of language and developmental abilities. Journal of Autism and Developmental Disorders 45, 3537–3549. 26100851 10.1007/s10803-015-2501-y PMC4609595

[ref31] Idring S, Rai D, Dal H, Dalman C, Sturm H, Zander E, Lee BK, Serlachius E and Magnusson C (2012) Autism spectrum disorders in the Stockholm Youth Cohort: design, prevalence and validity. PLoS One 7, e41280. 22911770 10.1371/journal.pone.0041280 PMC3401114

[ref32] Irwing P (2012) Sex differences in *g*: an analysis of the US standardization sample of the WAIS-III. Personality and Individual Differences 53, 126–131.

[ref33] Keller R, Chieregato S, Bari S, Castaldo R, Rutto F, Chiocchetti A and Dianzani U (2020) Autism in adulthood: clinical and demographic characteristics of a cohort of five hundred persons with autism analyzed by a novel multistep network model. Brain Sciences 10, 416. 10.3390/brainsci10070416 PMC7407178 32630229

[ref34] Kirkovski M, Enticott PG and Fitzgerald PB (2013) A review of the role of female gender in autism spectrum disorders. Journal of Autism and Developmental Disorders 43, 2584–2603. 23525974 10.1007/s10803-013-1811-1

[ref35] Lai M-C, Lombardo MV, Pasco G, Ruigrok AN, Wheelwright SJ, Sadek SA, Chakrabarti B, MRC AIMS Consortium and Baron-Cohen S (2011) A behavioral comparison of male and female adults with high functioning autism spectrum conditions. PLoS One 6, e20835. 21695147 10.1371/journal.pone.0020835 PMC3113855

[ref36] Longman RS, Saklofske DH and Fung TS (2007) WAIS-III percentile scores by education and sex for US and Canadian populations. Assessment 14, 426–432. 17986660 10.1177/1073191107304114

[ref37] Loomes R, Hull L and Mandy WPL (2017) What is the male-to-female ratio in autism spectrum disorder? A systematic review and meta-analysis. Journal of the American Academy of Child and Adolescent Psychiatry 56, 466–474. 28545751 10.1016/j.jaac.2017.03.013

[ref38] Lord C, Rutter M, DiLavore PC and Risi S (2002) Autism Diagnostic Observation Schedule. Los Angeles: Western Psychological Services.

[ref39] Maenner MJ, Shaw KA and Baio J (2016) Prevalence of autism spectrum disorder among children aged 8 years – autism and developmental disabilities monitoring network, 11 sites, United States, 2016. MMWR Surveillance Summaries 69, 1–12. 10.15585/mmwr.ss6904a1 PMC7119644 32214087

[ref40] Mandy W, Chilvers R, Chowdhury U, Salter G, Seigal A and Skuse D (2012) Sex differences in autism spectrum disorder: evidence from a large sample of children and adolescents. Journal of Autism and Developmental Disorders 42, 1304–1313. 21947663 10.1007/s10803-011-1356-0

[ref41] Mattila ML, Kielinen M, Linna SL, Jussila K, Ebeling H, Bloigu R, Joseph RM and Moilanen I (2011) Autism spectrum disorders according to DSM-IV-TR and comparison with DSM-5 draft criteria: an epidemiological study. Journal of the American Academy of Child and Adolescent Psychiatry 50, 583–592.e11. 21621142 10.1016/j.jaac.2011.04.001

[ref42] Metz CE (1978) Basic principles of ROC analysis. Seminars in Nuclear Medicine 8, 283–298. 112681 10.1016/s0001-2998(78)80014-2

[ref43] Mottron L (2004) Matching strategies in cognitive research with individuals with high-functioning autism: current practices, instrument biases, and recommendations. Journal of Autism and Developmental Disorders 34, 19–27. 15098953 10.1023/b:jadd.0000018070.88380.83

[ref44] Orsini A and Pezzuti L (2013) WAIS-IV. Contributo alla taratura italiana (16–69) (WAIS-IV, Contribution to the Italian Standardization, Ages 16–69). Firenze: Giunti OS.

[ref45] Pezzuti L, Lauriola M, Borella E, De Beni R and Cornoldi C (2019) Working Memory and Processing Speed mediate the effect of age on a General Ability Construct: evidence from the Italian WAIS-IV standardization sample. Personality and Individual Differences 138, 298–304.

[ref46] Pezzuti L, Tommasi M, Saggino A, Dawe J and Lauriola M (2020) Gender differences and measurement bias in the assessment of adult intelligence: evidence from the Italian WAIS-IV and WAIS-R standardizations. Intelligence 79, 101436.

[ref47] Raven J, Raven JC and Court JH (1998) Raven Manual: Section 4, Advanced Progressive Matrices, 1998 Edition. Oxford, UK: Oxford Psychologists Press Ltd.

[ref48] Ritvo RA, Ritvo ER, Guthrie D, Ritvo MJ, Hufnagel DH, McMahon W, Tonge B, Mataix-Cols D, Jassi A, Attwood T and Eloff J (2011) The Ritvo Autism Asperger Diagnostic Scale-Revised (RAADS-R): a scale to assist the diagnosis of autism spectrum disorder in adults: an international validation study. Journal of Autism and Developmental Disorders 41, 1076–1089. 21086033 10.1007/s10803-010-1133-5 PMC3134766

[ref49] Robert C, Borella E, Fagot D, Lecerf T and de Ribaupierre A (2009) Working memory and inhibitory control across the life span: intrusion errors in the Reading Span Task. Memory & Cognition 37, 336–345. 19246348 10.3758/MC.37.3.336

[ref50] Roid G (2003) Stanford-Binet Intelligence Scales, 5th Edn. Chicago, IL: Riverside.

[ref51] Roid GH, Miller LJ, Pomplun M and Koch C (2013) Leiter International Performance Scale, 3rd Edn. Wood dale, IL: Stoelting.

[ref52] R Studio Team (2020) RStudio: Integrated Development for R. RStudio. Boston, MA: PBC.

[ref53] Rutter M, Le Couteur A and Lord C (2003) Autism Diagnostic Interview–Revised. Los Angeles, CA: Western Psychological Services.

[ref54] Schaie KW and Willis SL (2010) The Seattle Longitudinal Study of adult cognitive development. ISSBD Bulletin 57, 24–29. 23536918 PMC3607395

[ref55] Siegel D, Minshew N and Goldstein G (1996) Wechsler IQ profiles in diagnosis of high-functioning autism. Journal of Autism and Developmental Disorders 26, 389–406. 8863091 10.1007/BF02172825

[ref56] Soulières I, Dawson M, Gernsbacher MA and Mottron L (2011) The level and nature of autistic intelligence II: what about Asperger syndrome? PLoS One 6, e25372. 21991394 10.1371/journal.pone.0025372 PMC3182210

[ref57] The Jamovi Project (2021) *Jamovi* (Version 1.6) [Computer Software]. Available at https://www.jamovi.org.

[ref58] Tommasi M, Watkins M, Orsini A, Pezzuti L, Cianci L and Saggino A (2015) Gender differences in latent cognitive abilities and education links with *g* in Italian elders. Learning and Individual Differences 37, 276–282.

[ref59] Van der Sluis S, Posthuma D, Dolan CV, de Geus EJC, Colom R and Boomsma DI (2006) Sex differences on the Dutch WAIS-III. Intelligence 34, 273–289.

[ref60] Van Wijngaarden-Cremers PJM, Van Eeten E, Groen WB, Van Deurzen PA, Oosterling IJ and Van der Gaag RJ (2014) Gender and age differences in the core triad of impairments in autism spectrum disorders: a systematic review and meta-analysis. Journal of Autism and Developmental Disorders 44, 627–635. 23989936 10.1007/s10803-013-1913-9

[ref61] Wechsler D (2013) Wechsler Adult Intelligence Scale – 4a Ed. (WAIS-IV), Italian Adaptation. Florence: Giunti O.S.

[ref62] Williams DL, Goldstein G, Kojkowski N and Minshew NJ (2008) Do individuals with high functioning autism have the IQ profile associated with nonverbal learning disability? Research in Autism Spectrum Disorders 2, 353–361. 18516234 10.1016/j.rasd.2007.08.005 PMC2394183

[ref63] Wilson CE, Murphy CM, McAlonan G, Robertson DM, Spain D, Hayward H, Woodhouse E, Deeley PQ, Gillan N, Ohlsen JC, Zinkstok J, Stoencheva V, Faulkner J, Yildiran H, Bell V, Hammond N, Craig MC and Murphy DG (2016) Does sex influence the diagnostic evaluation of autism spectrum disorder in adults? Autism 20, 808–819. 26802113 10.1177/1362361315611381 PMC5363500

[ref64] Zablotsky B, Black LI, Maenner MJ, Schieve LA and Blumberg SJ (2015) Estimated prevalence of autism and other developmental disabilities following questionnaire changes in the 2014 National Health Interview Survey. National Health Statistics Reports 87, 1–20. 26632847

[ref65] Zweig MH and Campbell G (1993) Receiver-operating characteristic (ROC) plots: a fundamental evaluation tool in clinical medicine. Clinical Chemistry 39, 561–577. 8472349

